# The regulation of the cell wall by glycosylphosphatidylinositol-anchored proteins in *Arabidopsis*

**DOI:** 10.3389/fcell.2022.904714

**Published:** 2022-08-12

**Authors:** Ke Zhou

**Affiliations:** Department of Biology, Pennsylvania State University, University Park, PA, United States

**Keywords:** cell wall, cellulose, pectin, glycosyl phosphatidylinositol, GPI-anchored protein

## Abstract

A polysaccharides-based cell wall covers the plant cell, shaping it and protecting it from the harsh environment. Cellulose microfibrils constitute the cell wall backbone and are embedded in a matrix of pectic and hemicellulosic polysaccharides and glycoproteins. Various environmental and developmental cues can regulate the plant cell wall, and diverse glycosylphosphatidylinositol (GPI)-anchored proteins participate in these regulations. GPI is a common lipid modification on eukaryotic proteins, which covalently tethers the proteins to the membrane lipid bilayer. Catalyzed by a series of enzymic complexes, protein precursors are post-translationally modified at their hydrophobic carboxyl-terminus in the endomembrane system and anchored to the lipid bilayer through an oligosaccharidic GPI modification. Ultimately, mature proteins reach the plasma membrane *via* the secretory pathway facing toward the apoplast and cell wall in plants. In *Arabidopsis*, more than three hundred GPI-anchored proteins (GPI-APs) have been predicted, and many are reported to be involved in diverse regulations of the cell wall. In this review, we summarize GPI-APs involved in cell wall regulation. GPI-APs are proposed to act as structural components of the cell wall, organize cellulose microfibrils at the cell surface, and during cell wall integrity signaling transduction. Besides regulating protein trafficking, the GPI modification is potentially governed by a GPI shedding system that cleaves and releases the GPI-anchored proteins from the plasma membrane into the cell wall.

## GPI-anchored proteins in *Arabidopsis*

The GPI is a common and conserved lipid modification on eukaryotic proteins (up to 0.5% of total proteins), which covalently links the protein to the lipid bilayer through an oligosaccharide structure (Oxley and Bacic, 1999). Eukaryotes share a conserved GPI biosynthesis mechanism better revealed in mammalian cells. The GPI moiety’s biosynthesis starts with a lipid molecule modification at the endoplasmic reticulum’s rough side (ER), then the modified lipid molecule flips into the luminal side of the ER. Catalyzed by a series of enzyme complexes, an oligosaccharide structure consisting of a minimal backbone of three mannoses (Mans), one non-N-acetylated glucosamine (GlcN), and inositol phospholipid, is added onto the modified lipid molecule (Stevens, 1995; Oxley and Bacic, 1999; Kinoshita and Fujita, 2016). A typical GPI-AP precursor possesses a secretory signal at the amino-terminus (N-terminus) that leads it to the ER lumen and a unique hydrophobic domain at the carboxyl-terminus (C-terminus) that is recognized while entering the ER lumen. Through a series of catalytic processes, the C-terminal hydrophobic region is hydrolyzed at the omega site, and an ethanolamine phosphate bond with the mannose of the GPI oligosaccharide structure replaces the peptide bond, connecting the GPI moiety covalently to the protein and with the membrane bilayers (Eisenhaber et al., 1998; Kinoshita, 2014; Kinoshita and Fujita, 2016). *via* an ADP-ribosylation factor (ARF)-mediated protein sorting system, GPI-APs apically target the sterol-rich microdomains in the plasma membrane of mammalian cells and expose the mature proteins to the external environment (Mayor and Riezman, 2004; Legler et al., 2005; Puig et al., 2019; Liu et al., 2021). Instead of being permanently tethered to lipid bilayers, GPI-APs could be released from the plasma membrane due to the cleavage catalyzed by the GPI-specific phospholipase (GPI-PLC) at the phospholipid phosphatidylinositol of the GPI oligosaccharide structure (Or ihashi et al., 2012; Fujihara and Ikawa, 2016).

A similar GPI biosynthesis system has been described in plant cells (Beihammer et al., 2020). Interrupting the first mannosylation step of GPI moiety biosynthesis is lethal for the plant and significantly affects the cell wall composition (Gillmor et al., 2005), and loss of function of the protease that hydrolyzes their precursors at the omega domain and transfers an assembled GPI anchor to mature protein causes global and severe developmental defections (Bundy et al., 2016). These reports conclude that GPI-APs are essential for plant growth, and their GPI modification is necessary for their function. However, disturbed remodeling of the GPI modification of GPI-APs significantly affects their efficient transport and correct cellular localization without causing severe developmental defections (Bernat-Silvestre et al., 2022), which indicates that GPI anchoring plays a role in their sorting. Nevertheless, the GPI shedding system identified in mammalians has not yet been found in plants. Plant GPI-APs have been best characterized in the model plant *Arabidopsis*, from which more than three hundred proteins are identified or predicted to possess GPI modification (Borner et al., 2002; Borner et al., 2003; Eisenhaber et al., 2003; Elortza et al., 2003). Previously, their participation in cell surface signaling transduction (Zhou, 2019a) and plasma membrane-cell wall nexus (Yeats et al., 2018) have been well-reviewed. Different from mammalian cells, GPI-APs tethering to the plasma membrane face toward the cell wall in plants, and many are documented to regulate the cell wall in *Arabidopsis*. Therefore, these cell wall-related GPI-APs are reviewed, and their mechanisms are discussed.

## The GPI-anchored Arabinogalactan Proteins

**FIGURE 1:**
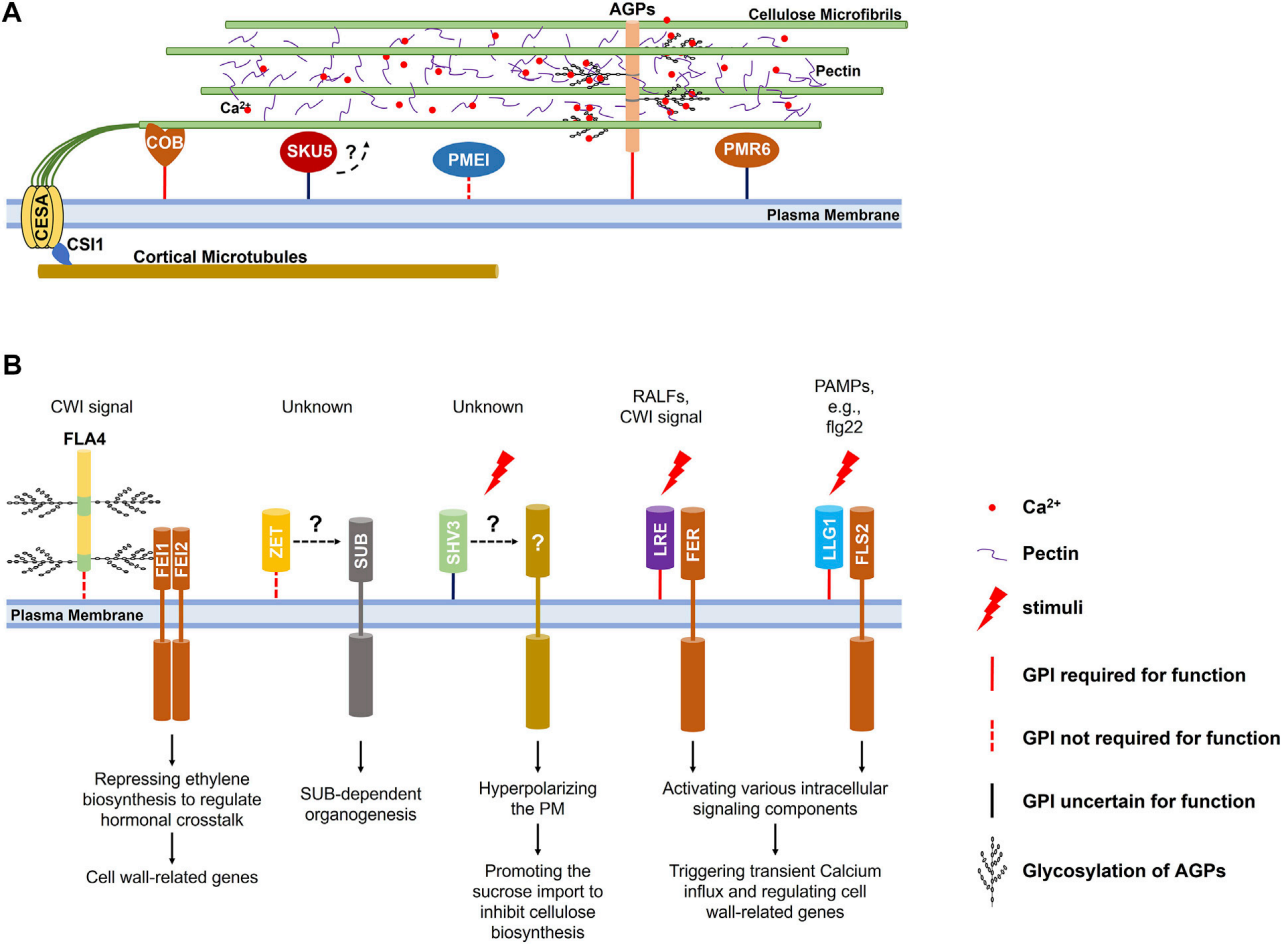
Regulations of the cell wall by GPI-APs in *Arabidopsis*. **(A)** Proposed model of GPI-APs acting to regulate cell wall structure. Besides acting as the structural protein that associates with cell wall polysaccharides to mediate cross-linking of cell wall components and the plasma membrane-cell wall integration, classical AGPs could accumulate and release calcium ions in a pH-dependent manner to regulate pectin cross-linking; COB and its homologs participate in the alignment of cortical microtubules and cellulose microfibrils; PMEI1 and PMR6 are involved in esterification and metabolism of pectin; SKU5 and its homologs are crucial for anisotropic cell expansion through regulating cell wall structure under an unidentified mechanism. **(B)** GPI-APs suggested to function in cell wall signaling pathways. FLA4 acts as a ligand relating to damaged cell wall integrity and participates in FEI1/2 mediated signaling transduction; ZET and ZETH might contribute to the non-cell-autonomous SUB-mediated signaling transduction; SHV3 and SVL1 might participate in signaling transduction during cell expansion and coordinate the proton pumping and the cellulose synthesis; LRE and its homologs chaperone transmembrane receptors to recognize various ligands. The GPI anchor of these GPI-APs is a solid red line when it is essential for function, a dotted red line when functionally dispensable, and a solid black line when unidentified.

Highly glycosylated (>90% of total molecular mass), hydroxyproline-rich arabinogalactan proteins (AGPs) might be the most complex and diverse glycoprotein family in plants (Chasan, 1994). A classical AGP is modified by a GPI anchor and glycosylated by N- and O-glycosylation at Pro-Ala-Ser-Thr (PAST) repeats where complex and heterogenous arabinogalactan glycans are added (Shpak et al., 1999; Ellis et al., 2010; Duruflé et al., 2017; Ma et al., 2017). As the primary structural glycoprotein components of the plant cell wall, GPI-anchored AGPs have been well-reviewed recently (Silva et al., 2020; Hromadová et al., 2021). GPI-anchored AGPs participate in cross-linking of cell wall components through non-covalent (Lamport et al., 2005; Hijazi et al., 2014) or covalent (Tan et al., 2013) association between their glycan modifications and the hemicellulosic and pectic polysaccharides. Attributing to the GPI anchoring to the plasma membrane, the cross-linking with polysaccharidic components also allows AGPs to mediate the plasma membrane-cell wall integration (Palacio-Lopez et al., 2019) (Figure 1A).

Apoplastic calcium ions participate in the formation of an “egg-box” structure that enhances the cross-linking of pectic polysaccharides in the cell wall matrix (Lampugnani et al., 2018). Glycan modifications of GPI-anchored AGPs could also act as capacitors that accumulate calcium ions in the cell wall matrix through their glucuronic acid (GlcA) carboxyls in a pH-dependent manner. Calcium ions are discharged into the cell wall matrix in a low pH environment, which could be supplied for pectin cross-linking and calcium ions influx (Lamport and Varnai, 2013; Lope z-Hernandez et al., 2020) (Figure 1A).

Besides classical AGPs, some chimeric Fasciclin-Like AGPs (FLAs) are also modified by GPI anchors, among which FLA4 (SOS5) is the most extensively studied for its participation in leucine-rich receptor-like kinases (LRR-RLKs) FEI1/2-mediated cell wall integrity (CWI) signaling pathway (Seifert, 2021). Apart from localizing to the plasma membrane, FLA4 could be released into the apoplast. Interestingly, GPI anchoring is required for the efficient transport to the plasma membrane *via* the secretory pathway, but not the function (Griffiths et al., 2016; Xue et al., 2017). Although no direct experimental evidence supports the interaction between FLA4 and FEI1/2, FLA4 is considered to act as a regulatory ligand that exhibits the CWI-related signal to activate a receptor complex containing FEI1/2 (Figure 1B). In this process, the glycosylation of FLA4 catalyzed by galactosyltransferases (GALT) is required (Basu et al., 2016). The activated receptor complex leads to hormonal and developmental alterations, consequently regulating cell wall-related genes (Seifert et al., 2014; Acet and Kadioglu, 2020; Seifert, 2021). Loss of function of *FLA4* results in defected anisotropic cell expansion, thinner cell wall and affected cell adhesion in root (Shi et al., 2003), and decreased cellulose deposit in seed mucilage (Harpaz-Saad et al., 2012). Interestingly, its GPI-anchored homologs, FLA11 and FLA12, are also supposed to be involved in CWI-related signal complexes that sense the mechanical stimuli and initiate the development of the secondary cell wall (Ma et al., 2022). Nevertheless, how they work as ligands relating to the CWI-related signal remains largely unknown.

## GPI-anchored SHV3 and SVL1

Cellulose constitutes the backbone of the plant cell wall is built up by Uridine-5′-Diphosphate-Glucose (UDP-Glc) (Polko and Kieber, 2019). Generally, newly fixed carbon by photosynthesis is converted into sucrose, transported to sink organs *via* the phloem, and imported into the cytoplasm. In the cytoplasm, sucrose is cleaved into UDP-Glc and fructose by Sucrose Synthase (SUS) (Verbancic et al., 2018). Then UDP-Glc molecules are converted into β-(1→4)-D-glucan chains by the trans-plasma membrane Cellulose Synthase (CESA) subunits of the cellulose synthase complexes (CSCs) at the cell surface (McFarlane et al., 2014). In *Arabidopsis* root, cytoplasmic sucrose imported *via* a plasma membrane-localized Suc/H^+^ symporter is one of the significant regulators of cellulose biosynthesis, and the excessive cytoplasmic sucrose reduces the velocity of CSCs on the plasma membrane, and the cellulose biosynthesis through an unrevealed mechanism (Li et al., 2014; Verbancic et al., 2018). *SHV3* and its homolog *SVL1* encode GPI-anchored glycerophosphodiester phosphodiesterase-like (GPDL) proteins. When exogenous sucrose is provided in the medium, loss of functions of *SHV3* and *SVL1* significantly promotes the sucrose importation to inhibit the cellulose biosynthesis in the root, which could be suppressed by the loss of function of plasma membrane-localized Suc/H^+^ symporter (Yeats et al., 2016; Yeats and Somerville, 2016). The hyperpolarized plasma membrane H^+^ gradient of *shv3/svl1* mutant root seems responsible for the excessive sucrose importation, which is also reported in the loss of function of a transmembrane Receptor-Like Kinase (RLK) FERONIA (FER) that mediates various cell wall-related singling transduction and response (Feng et al., 2018). It implies the involvement of SHV3 and SVL1 in signaling transduction during cell expansion that coordinates the proton pumping and the cellulose synthesis (Figure 1B). However, it is still unclear whether they chaperone relative transmembrane signaling transduction or act as ligands.

## GPI-anchored COBRAs

Due to the association between CSCs and cortical microtubules beneath the plasma membrane through Cellulose Synthase Interactive 1 (CSI1), the synthesized cellulosic chains assemble microfibrils that align with the cortical microtubule arrays (Baskin and Gu, 2012; Watanabe et al., 2015; Bidhendi et al., 2020). Through hydrogen bonds and Van der Waals forces, the synthesized cellulosic chains assemble microfibrils that align with the cortical microtubule arrays (Cosgrove, 2014; Kubicki et al., 2018; Zhang et al., 2021). The aligned cortical microtubule and cellulose microfibril arrays determine the anisotropic cell expansion (Sedbrook and Kaloriti, 2008; Crowell et al., 2010). *COBRA* (*COB*) and its homologs encode GPI-anchored proteins that possess active cellulose-binding domains (Roudier et al., 2002). COB predominately localizes on the plasma membrane and in the cell wall of the longitudinal sides of rapidly elongating root cells. COB on the plasma membrane aligns with the cortical microtubules, and its loss of function severely affects the anisotropic cell expansion in various organs (Schindelman et al., 2001; Roudier et al., 2002; Ko et al., 2006; Liu et al., 2013), primarily due to the disorientated cellulose microfibrils (Roudier et al., 2005). These results imply that COB may play a central role in a hypothesized complex that aligns the cellulose microfibrils with cortical microtubules (Figure 1A). Homologs of the COB are also reportedly involved in cellulose deposition in root hair tips (Li et al., 2022) and seed mucilage (Ben-Tov et al., 2018), as well as in cell-cell communication during fertilization (Li et al., 2013), but whether these involvements originate from affected cellulose microfibrils’ orientation or other mechanisms requires further investigations.

## GPI-anchored PMEs and PMEIs

Pectin is a major polysaccharidic component biosynthesized in the endoplasmic membrane system and secreted into the cell wall matrix to provide structural support, cross-link cell wall components, mediate cell wall adhesion, and supply cell-related signal molecules (Ridley et al., 2001; Lehner et al., 2010; Cao, 2012; Peaucelle et al., 2012; Daher and Braybrook, 2015; Sista Kameshwar and Qin, 2018). The de-esterification at the C6 atom of galacturonic acid (GalA) of the homogalacturonan (HG) backbone is required for binding calcium ions to form an “egg-box” structure that cross-links and stiffens pectin (Lampugnani et al., 2018). The de-esterification is catalyzed by the pectin methylesterases (PMEs), of which activity could be inhibited by the pectin methylesterase inhibitors (PMEIs) (Peaucelle et al., 2012; Wormit and Usadel, 2018). Several PMEs and PMEIs are predicted to be GPI-anchored proteins through informatics and proteomic assays (Takahashi et al., 2016). Still, only PMEI1 is confirmed by experimental data, of which GPI anchoring is crucial for its secretion to the cell wall matrix where it functions (De Caroli et al., 2011) (Figure 1A). PMEI1 is supposed to be functional in the cell wall matrix to inhibit the PME activity, and it does partially localize there, which implies its release from the plasma membrane. Whether the release originates from the cleavage of GPI anchoring is unclear.

## GPI-anchored PMR6

Pectate lyases (PLs) and Pectate lyase-like proteins (PLLs) are reported to be involved in pectin degradation and potentially release of pectic oligosaccharides for cell wall-related signaling response (Palusa et al., 2007; Cao, 2012; Chen et al., 2021). Among 27 predicted pectate lyase–like members in *Arabidopsis*, PMR6 is the only one modified by the GPI anchoring. PMR6 is required for general plant growth and powdery mildew susceptibility. Its loss of function alters the composition of the plant cell wall and exhibits higher resistance to powdery mildew (Vogel et al., 2002). It is hypothesized that PMR6 is required to promote fungal growth, or the loss of function of *PMR6* activates specific defenses response inhibiting fungal infection (Figure 1A). Nevertheless, we know very little about PMR6 and its GPI anchoring.

## GPI-anchored LORELEI and LLGs

Chaperoning extracellular domains of transmembrane Receptor-like Kinases to recognize extracellular signal molecules at the cell surface is one of the typical functions of GPI-APs (Zhou, 2019a). Not only chaperoning the delivery of FER to the plasma membrane, the homolog of GPI-anchored LORELEI (LRE) LLG1 associates with the extracellular domain of FER to form a receptor complex at the cell surface to recognize the signaling polypeptides RALF23 (Xiao et al., 2019). They trigger a rapid apoplastic alkalization that could significantly inhibit the expansin-dependent acidic cell wall expansion (Cosgrove, 2005). Besides the signaling polypeptides, the LRE/LLG1-FER complex could recognize the pectic signal originating from damaged cell wall integrity and trigger a calcium-dependent cell wall rescue (Duan et al., 2010; Feng et al., 2018). Interestingly, LLG1 also chaperones a membrane-localized pattern recognition receptors (PRRs) FLS2 to recognize pathogen-associated molecular patterns (PAMPs) and to trigger the transient calcium ions influx, and Mitogen-Activated Protein Kinase (MAPK) cascades (Shen et al., 2017; Chi et al., 2021) (Figure 1B). These reports imply that LRE and its three homologs may combine with different RLKs to recognize diverse ligands.

## GPI-anchored ZET and ZETH

GPI-anchored ZERZAUST (ZET) and its homolog ZETH contribute to organogenesis that relies on a trans-plasma membrane receptor STRUBBELIG (SUB) (Fulton et al., 2009). SUB is a non-cell-autonomous atypical Receptor-Like Kinase that controls tissue morphogenesis and responses to damaged cell wall integrity. Interestingly, the intracellular kinase domain is not required for its functions (Cha udhary et al., 2020; Chaudhary et al., 2021). Loss of function of *ZET* results in severe defection in the outer integuments development, floral organs morphogenesis, and stems anisotropic elongation, for which altered cell wall composition and structure might be responsible (Vaddepalli et al., 2017). ZET predominantly localizes longitudinally within the apoplast of lamella junctions in a non-cell-autonomous fashion and can spread locally through the apoplast. GPI anchoring is dispensable for its localization and function but is required for an efficient transport (Fulton et al., 2009; Vaddepalli et al., 2017; Vaddepalli et al., 2019) (Figure 1B). The apoplastic localization and dispensability of GPI anchoring imply that ZET might act as a ligand that exhibits the damaged cell wall integrity rather than chaperone SUB-mediated signaling transduction. However, we know very little about this.

## GPI-anchored SKU5 and SKSs

Organ spiral growth ubiquitously exists in the plant world, and *Arabidopsis* roots exhibit slight left-handedness that originates from naturally inclined root cell elongation (Smyth, 2016; Buschmann and Borchers, 2020). Besides cortical microtubule arrays (Furutani et al., 2000; Thitamadee et al., 2002; Ishida et al., 2007; Galva et al., 2014), root spiral growth of *Arabidopsis* has also been reported to be related to the cell wall (Buschmann and Borchers, 2020). *SKU5* is expressed in rapid-growing organs and encodes a GPI-anchored protein structurally related to the multiple-copper oxidases. In the root, SKU5 could be found in the cell wall and attached to the plasma membrane but mainly accumulated longitudinally in lamella junctions of the epidermis and cortex at the elongation zone. Possibly due to the slightly reduced elongation rate of root epidermal cells compared with inner layers, the root’s natural left-handedness is aggravated in *sku5* mutant (Sedbrook et al., 2002). Further investigations on the loss of functions of *SKU5* and its homologs conclude their involvement in the anisotropic expansion of root epidermal cells, possibly through regulating cell wall structure (Zhou, 2019b). Nevertheless, the regulation of cell wall structure by SKU5 and its homologs and the importance of GPI anchoring in this process are still largely unknown (Figure 1A).

## Discussions

Mature GPI-APs are anchored to the plasma membrane and exposed to the cell wall matrix in plant cells, which endows them with unique features in regulating the cell wall. This review summarizes the broad involvements of GPI-APs in the cell wall regulations in *Arabidopsis*, including acting as structural glycoproteins of the cell wall, regulating cellulose biosynthesis and cellulose microfibrils orientation, controlling pectin cross-linking by providing calcium, handling pectin esterification and metabolism, and serving as ligand or co-receptor to mediate cell wall-related signaling transduction.

In Mammalian cells, besides covalently tethering GPI-APs to the lipid bilayer, the GPI modification acts as a sorting signal that apically drives GPI-APs to cholesterol-rich microdomains in the plasma membrane *via* an ARF-mediated secretory pathway (Puig et al., 2019). In *Arabidopsis*, a similar role of the GPI anchor has also been identified in FLA4, PMEI1, ZET, COBL10, and LRE/LLGs. However, the GPI-mediated sorting system and its contribution to cell wall regulation are largely unknown in plants.

Although the GPI-specific phospholipase that cleaves the GPI anchor and releases the GPI-APs from the plasma membrane has not been identified in plants, experimental evidence supports its existence. Most GPI-APs summarized in this review are found partially localized in the cell wall matrix besides attached to the plasma membrane. For those GPI-APs supposed to be functional in the apoplast or the cell wall matrix, such as FLA4, PMEI1, and ZET, GPI anchoring is not required for their function. In contrast, GPI anchoring is required for those GPI-APs supposed to be functional at the plasma membrane, such as LRE/LLGs (Li et al., 2015) and COB/COBLs. It implies an unidentified GPI shedding system that regulates the alteration of GPI-APs from plasma membrane-associated proteins to apoplastic or cell wall proteins. However, we know very little about it.

## References

[B1] Acet T. Kadioglu A. (2020). SOS5 gene-abscisic acid crosstalk and their interaction with antioxidant system in *Arabidopsis thaliana* under salt stress. Physiol. Mol. Biol. Plants 26, 1831–1845. 10.1007/s12298-020-00873-4 32943819 PMC7468026

[B2] Baskin T. I. Gu Y. (2012). Making parallel lines meet: Transferring information from microtubules to extracellular matrix. Cell adh. Migr. 6, 404–408. 10.4161/cam.21121 22902763 PMC3496676

[B3] Basu D. Tian L. Debrosse T. Poirier E. Emch K. Herock H. (2016). Glycosylation of a fasciclin-like arabinogalactan-protein (SOS5) mediates root growth and seed mucilage adherence via a cell wall receptor-like kinase (FEI1/FEI2) pathway in Arabidopsis. PLoS One 11, e0145092. 10.1371/journal.pone.0145092 26731606 PMC4701510

[B4] Beihammer G. Maresch D. Altmann F. Strasser R. (2020). Glycosylphosphatidylinositol-anchor synthesis in plants: A glycobiology perspective. Front. Plant Sci. 11, 611188. 10.3389/fpls.2020.611188 33312189 PMC7704450

[B5] Ben-Tov D. Idan-Molakandov A. Hugger A. Ben-Shlush I. Gunl M. Yang B. (2018). The role of COBRA-LIKE 2 function, as part of the complex network of interacting pathways regulating Arabidopsis seed mucilage polysaccharide matrix organization. Plant J. 94, 497–512. 10.1111/tpj.13871 29446495

[B6] Bernat-Silvestre C. Ma Y. Johnson K. Ferrando A. Aniento F. Marcote M. J. (2022). Characterization of Arabidopsis post-glycosylphosphatidylinositol attachment to proteins phospholipase 3 like genes. Front. Plant Sci. 13, 817915. 10.3389/fpls.2022.817915 35222477 PMC8874281

[B7] Bidhendi A. J. Chebli Y. Geitmann A. (2020). Fluorescence visualization of cellulose and pectin in the primary plant cell wall. J. Microsc. 278, 164–181. 10.1111/jmi.12895 32270489

[B8] Borner G. H. Lilley K. S. Stevens T. J. Dupree P. (2003). Identification of glycosylphosphatidylinositol-anchored proteins in Arabidopsis. A proteomic and genomic analysis. Plant Physiol. 132, 568–577. 10.1104/pp.103.021170 12805588 PMC166998

[B9] Borner G. H. Sherrier D. J. Stevens T. J. Arkin I. T. Dupree P. (2002). Prediction of glycosylphosphatidylinositol-anchored proteins in Arabidopsis. A genomic analysis. Plant Physiol. 129, 486–499. 10.1104/pp.010884 12068095 PMC161667

[B10] Bundy M. G. Kosentka P. Z. Willet A. H. Zhang L. Miller E. Shpak E. D. (2016). A mutation in the catalytic subunit of the glycosylphosphatidylinositol transamidase disrupts growth, fertility, and stomata formation. Plant Physiol. 171, 974–985. 10.1104/pp.16.00339 27208238 PMC4902618

[B11] Buschmann H. Borchers A. (2020). Handedness in plant cell expansion: A mutant perspective on helical growth. New Phytol. 225, 53–69. 10.1111/nph.16034 31254400

[B12] Cao J. (2012). The pectin lyases in *Arabidopsis thaliana*: Evolution, selection and expression profiles. PLoS One 7, e46944. 10.1371/journal.pone.0046944 23056537 PMC3467278

[B13] Chaudhary A. Chen X. Gao J. Lesniewska B. Hammerl R. Dawid C. (2020). The Arabidopsis receptor kinase STRUBBELIG regulates the response to cellulose deficiency. PLoS Genet. 16, e1008433. 10.1371/journal.pgen.1008433 31961852 PMC6994178

[B14] Chasan R. (1994). Arabinogalactan proteins - getting to the core. Plant Cell 6, 1519. 10.2307/3869938

[B15] Chaudhary A. Chen X. Lesniewska B. Boikine R. Gao J. Wolf S. (2021). Cell wall damage attenuates root hair patterning and tissue morphogenesis mediated by the receptor kinase STRUBBELIG. Development 148, dev199425. 10.1242/dev.199425 34251020

[B16] Chen Y. Li W. Turner J. A. Anderson C. T. (2021). PECTATE LYASE LIKE12 patterns the guard cell wall to coordinate turgor pressure and wall mechanics for proper stomatal function in Arabidopsis. Plant Cell 33, 3134–3150. 10.1093/plcell/koab161 34109391 PMC8462824

[B17] Chi Y. Wang C. Wang M. Wan D. Huang F. Jiang Z. (2021). Flg22-induced Ca(2+) increases undergo desensitization and resensitization. Plant Cell Environ. 44, 3563–3575. 10.1111/pce.14186 34536020

[B18] Cosgrove D. J. (2005). Growth of the plant cell wall. Nat. Rev. Mol. Cell Biol. 6, 850–861. 10.1038/nrm1746 16261190

[B19] Cosgrove D. J. (2014). Re-constructing our models of cellulose and primary cell wall assembly. Curr. Opin. Plant Biol. 22, 122–131. 10.1016/j.pbi.2014.11.001 25460077 PMC4293254

[B20] Crowell E. F. Gonneau M. Vernhettes S. Hofte H. (2010). Regulation of anisotropic cell expansion in higher plants. C. R. Biol. 333, 320–324. 10.1016/j.crvi.2010.01.007 20371106

[B21] Daher F. B. Braybrook S. A. (2015). How to let go: Pectin and plant cell adhesion. Front. Plant Sci. 6, 523. 10.3389/fpls.2015.00523 26236321 PMC4500915

[B22] De Caroli M. Lenucci M. S. Di Sansebastiano G. P. Dalessandro G. De Lorenzo G. Piro G. (2011). Protein trafficking to the cell wall occurs through mechanisms distinguishable from default sorting in tobacco. Plant J. 65, 295–308. 10.1111/j.1365-313X.2010.04421.x 21223393

[B23] Duan Q. Kita D. Li C. Cheung A. Y. Wu H. M. (2010). FERONIA receptor-like kinase regulates RHO GTPase signaling of root hair development. Proc. Natl. Acad. Sci. U. S. A. 107, 17821–17826. 10.1073/pnas.1005366107 20876100 PMC2955125

[B24] Duruflé H. Hervé V. Balliau T. Zivy M. Dunand C. Jamet E. (2017). Proline hydroxylation in cell wall proteins: Is it yet possible to define rules? Front. Plant Sci. 8, 1802. 10.3389/fpls.2017.01802 29089960 PMC5651053

[B25] Eisenhaber B. Bork P. Eisenhaber F. (1998). Sequence properties of GPI-anchored proteins near the omega-site: Constraints for the polypeptide binding site of the putative transamidase. Protein Eng. 11, 1155–1161. 10.1093/protein/11.12.1155 9930665

[B26] Eisenhaber B. Wildpaner M. Schultz C. J. Borner G. H. Dupree P. Eisenhaber F. (2003). Glycosylphosphatidylinositol lipid anchoring of plant proteins. Sensitive prediction from sequence- and genome-wide studies for Arabidopsis and rice. Plant Physiol. 133, 1691–1701. 10.1104/pp.103.023580 14681532 PMC300724

[B27] Ellis M. Egelund J. Schultz C. J. Bacic A. (2010). Arabinogalactan-proteins: Key regulators at the cell surface? Plant Physiol. 153, 403–419. 10.1104/pp.110.156000 20388666 PMC2879789

[B28] Elortza F. Nuhse T. S. Foster L. J. Stensballe A. Peck S. C. Jensen O. N. (2003). Proteomic analysis of glycosylphosphatidylinositol-anchored membrane proteins. Mol. Cell. Proteomics 2, 1261–1270. 10.1074/mcp.M300079-MCP200 14517339

[B29] Feng W. Kita D. Peaucelle A. Cartwright H. N. Doan V. Duan Q. (2018). The FERONIA receptor kinase maintains cell-wall integrity during salt stress through Ca(2+) signaling. Curr. Biol. 28, 666–675. 10.1016/j.cub.2018.01.023 29456142 PMC5894116

[B30] Fujihara Y. Ikawa M. (2016). GPI-AP release in cellular, developmental, and reproductive biology. J. Lipid Res. 57, 538–545. 10.1194/jlr.R063032 26593072 PMC4808780

[B31] Fulton L. Batoux M. Vaddepalli P. Yadav R. K. Busch W. Andersen S. U. (2009). DETORQUEO, QUIRKY, and ZERZAUST represent novel components involved in organ development mediated by the receptor-like kinase STRUBBELIG in *Arabidopsis thaliana* . PLoS Genet. 5, e1000355. 10.1371/journal.pgen.1000355 19180193 PMC2628281

[B32] Furutani I. Watanabe Y. Prieto R. Masukawa M. Suzuki K. Naoi K. (2000). The SPIRAL genes are required for directional control of cell elongation in Aarabidopsis thaliana. Development 127, 4443–4453. 10.1242/dev.127.20.4443 11003843

[B33] Galva C. Kirik V. Lindeboom J. J. Kaloriti D. Rancour D. M. Hussey P. J. (2014). The microtubule plus-end tracking proteins SPR1 and EB1b interact to maintain polar cell elongation and directional organ growth in Arabidopsis. Plant Cell 26, 4409–4425. 10.1105/tpc.114.131482 25415978 PMC4277225

[B34] Gillmor C. S. Lukowitz W. Brininstool G. Sedbrook J. C. Hamann T. Poindexter P. (2005). Glycosylphosphatidylinositol-anchored proteins are required for cell wall synthesis and morphogenesis in Arabidopsis. Plant Cell 17, 1128–1140. 10.1105/tpc.105.031815 15772281 PMC1087991

[B35] Griffiths J. S. Crepeau M. J. Ralet M. C. Seifert G. J. North H. M. (2016). Dissecting seed mucilage adherence mediated by FEI2 and SOS5. Front. Plant Sci. 7, 1073. 10.3389/fpls.2016.01073 27524986 PMC4965450

[B36] Harpaz-Saad S. Western T. L. Kieber J. J. (2012). The FEI2-SOS5 pathway and CELLULOSE SYNTHASE 5 are required for cellulose biosynthesis in the Arabidopsis seed coat and affect pectin mucilage structure. Plant Signal. Behav. 7, 285–288. 10.4161/psb.18819 22353871 PMC3405700

[B37] Hijazi M. Velasquez S. M. Jamet E. Estevez J. M. Albenne C. c. (2014). An update on post-translational modifications of hydroxyproline-rich glycoproteins: Toward a model highlighting their contribution to plant cell wall architecture. Front. Plant Sci. 5, 395. 10.3389/fpls.2014.00395 25177325 PMC4132260

[B38] Hromadová D. Soukup A. Tylová E. (2021). Arabinogalactan proteins in plant roots – an update on possible functions. Front. Plant Sci. 12, 674010. 10.3389/fpls.2021.674010 34079573 PMC8165308

[B39] Ishida T. Kaneko Y. Iwano M. Hashimoto T. (2007). Helical microtubule arrays in a collection of twisting tubulin mutants of *Arabidopsis thaliana* . Proc. Natl. Acad. Sci. U. S. A. 104, 8544–8549. 10.1073/pnas.0701224104 17488810 PMC1895986

[B40] Kinoshita T. (2014). Enzymatic mechanism of GPI anchor attachment clarified. Cell Cycle 13, 1838–1839. 10.4161/cc.29379 24865529 PMC4111747

[B41] Kinoshita T. Fujita M. (2016). Biosynthesis of GPI-anchored proteins: Special emphasis on GPI lipid remodeling. J. Lipid Res. 57, 6–24. 10.1194/jlr.R063313 26563290 PMC4689344

[B42] Ko J. H. Kim J. H. Jayanty S. S. Howe G. A. Han K. H. (2006). Loss of function of COBRA, a determinant of oriented cell expansion, invokes cellular defence responses in *Arabidopsis thaliana* . J. Exp. Bot. 57, 2923–2936. 10.1093/jxb/erl052 16873454

[B43] Kubicki J. D. Yang H. Sawada D. O'Neill H. Oehme D. Cosgrove D. (2018). The shape of native plant cellulose microfibrils. Sci. Rep. 8, 13983. 10.1038/s41598-018-32211-w 30228280 PMC6143632

[B44] Lamport D. T. A. Kieliszewski M. J. Showalter A. M. (2005). Salt stress upregulates periplasmic arabinogalactan proteins: Using salt stress to analyse AGP function. New Phytol. 169, 479–492. 10.1111/j.1469-8137.2005.01591.x 16411951

[B45] Lamport D. T. A. Varnai P. (2013). Periplasmic arabinogalactan glycoproteins act as a calcium capacitor that regulates plant growth and development. New Phytol. 197, 58–64. 10.1111/nph.12005 23106282

[B46] Lampugnani E. R. Khan G. A. Somssich M. Persson S. (2018). Building a plant cell wall at a glance. J. Cell Sci. 131, jcs207373. 10.1242/jcs.207373 29378834

[B47] Legler D. F. Doucey M. A. Schneider P. Chapatte L. Bender F. C. Bron C. (2005). Differential insertion of GPI-anchored GFPs into lipid rafts of live cells. FASEB J. 19, 73–75. 10.1096/fj.03-1338fje 15516372

[B48] Lehner A. Dardelle F. Soret-Morvan O. Lerouge P. Driouich A. Mollet J. C. (2010). Pectins in the cell wall of *Arabidopsis thaliana* pollen tube and pistil. Plant Signal. Behav. 5, 1282–1285. 10.4161/psb.5.10.13040 20861690 PMC3115368

[B49] Li C. Yeh F. L. Cheung A. Y. Duan Q. Kita D. Liu M. C. (2015). Glycosylphosphatidylinositol-anchored proteins as chaperones and co-receptors for FERONIA receptor kinase signaling in Arabidopsis. Elife 4. 10.7554/eLife.06587 PMC4458842 26052747

[B50] Li S. Bashline L. Lei L. Gu Y. (2014). Cellulose synthesis and its regulation. Arab. Book 12, e0169. 10.1199/tab.0169 PMC3894906 24465174

[B51] Li S. Ge F. R. Xu M. Zhao X. Y. Huang G. Q. Zhou L. Z. (2013). Arabidopsis COBRA-LIKE 10, a GPI-anchored protein, mediates directional growth of pollen tubes. Plant J. 74, 486–497. 10.1111/tpj.12139 23384085

[B52] Li Z. Zhou T. Sun P. Chen X. Gong L. Sun P. (2022). COBL9 and COBL7 synergistically regulate root hair tip growth via controlling apical cellulose deposition. Biochem. Biophys. Res. Commun. 596, 6–13. 10.1016/j.bbrc.2022.01.096 35104663

[B53] Liu L. Shang-Guan K. Zhang B. Liu X. Yan M. Zhang L. (2013). Brittle Culm1, a COBRA-like protein, functions in cellulose assembly through binding cellulose microfibrils. PLoS Genet. 9, e1003704. 10.1371/journal.pgen.1003704 23990797 PMC3749933

[B54] Liu S.-S. Liu Y.-S. Guo X.-Y. Murakami Y. Yang G. Gao X.-D. (2021). A knockout cell library of GPI biosynthetic genes for functional studies of GPI-anchored proteins. Commun. Biol. 4, 777. 10.1038/s42003-021-02337-1 34162996 PMC8222316

[B55] Lopez-Hernandez F. Tryfona T. Rizza A. Yu X. L. Harris M. O. B. Webb A. A. R. (2020). Calcium binding by arabinogalactan polysaccharides is important for normal plant development. Plant Cell 32, 3346–3369. 10.1105/tpc.20.00027 32769130 PMC7534474

[B56] Ma Y. MacMillan C. P. de Vries L. Mansfield S. D. Hao P. Ratcliffe J. (2022). FLA11 and FLA12 glycoproteins fine-tune stem secondary wall properties in response to mechanical stresses. New Phytol. 233, 1750–1767. 10.1111/nph.17898 34862967 PMC9302641

[B57] Ma Y. Yan C. Li H. Wu W. Liu Y. Wang Y. (2017). Bioinformatics prediction and evolution analysis of arabinogalactan proteins in the plant kingdom. Front. Plant Sci. 8, 66. 10.3389/fpls.2017.00066 28184232 PMC5266747

[B58] Mayor S. Riezman H. (2004). Sorting GPI-anchored proteins. Nat. Rev. Mol. Cell Biol. 5, 110–120. 10.1038/nrm1309 15040444

[B59] McFarlane H. E. Doring A. Persson S. (2014). The cell biology of cellulose synthesis. Annu. Rev. Plant Biol. 65, 69–94. 10.1146/annurev-arplant-050213-040240 24579997

[B60] Orihashi K. Tojo H. Okawa K. Tashima Y. Morita T. Kondoh G. (2012). Mammalian carboxylesterase (CES) releases GPI-anchored proteins from the cell surface upon lipid raft fluidization. Biol. Chem. 393, 169–176. 10.1515/hsz-2011-0269 22718632

[B61] Oxley D. Bacic A. (1999). Structure of the glycosylphosphatidylinositol anchor of an arabinogalactan protein from Pyrus communis suspension-cultured cells. Proc. Natl. Acad. Sci. U. S. A. 96, 14246–14251. 10.1073/pnas.96.25.14246 10588691 PMC24422

[B62] Palacio-Lopez K. Tinaz B. Holzinger A. Domozych D. S. (2019). Arabinogalactan proteins and the extracellular matrix of charophytes: A sticky business. Front. Plant Sci. 10, 447. 10.3389/fpls.2019.00447 31031785 PMC6474363

[B63] Palusa S. G. Golovkin M. Shin S. B. Richardson D. N. Reddy A. S. N. (2007). Organ-specific, developmental, hormonal and stress regulation of expression of putative pectate lyase genes in Arabidopsis. New Phytol. 174, 537–550. 10.1111/j.1469-8137.2007.02033.x 17447910

[B64] Peaucelle A. Braybrook S. Hofte H. (2012). Cell wall mechanics and growth control in plants: The role of pectins revisited. Front. Plant Sci. 3, 121. 10.3389/fpls.2012.00121 22685449 PMC3368173

[B65] Polko J. K. Kieber J. J. (2019). The regulation of cellulose biosynthesis in plants. Plant Cell 31, 282–296. 10.1105/tpc.18.00760 30647077 PMC6447023

[B66] Puig B. Altmeppen H. C. Linsenmeier L. Chakroun K. Wegwitz F. Piontek U. K. (2019). GPI-anchor signal sequence influences PrPC sorting, shedding and signalling, and impacts on different pathomechanistic aspects of prion disease in mice. PLoS Pathog. 15, e1007520. 10.1371/journal.ppat.1007520 30608982 PMC6334958

[B67] Ridley B. L. O'Neill M. A. Mohnen D. (2001). Pectins: Structure, biosynthesis, and oligogalacturonide-related signaling. Phytochemistry 57, 929–967. 10.1016/s0031-9422(01)00113-3 11423142

[B68] Roudier F. Fernandez A. G. Fujita M. Himmelspach R. Borner G. H. Schindelman G. (2005). COBRA, an Arabidopsis extracellular glycosyl-phosphatidyl inositol-anchored protein, specifically controls highly anisotropic expansion through its involvement in cellulose microfibril orientation. Plant Cell 17, 1749–1763. 10.1105/tpc.105.031732 15849274 PMC1143074

[B69] Roudier F. Schindelman G. DeSalle R. Benfey P. N. (2002). The COBRA family of putative GPI-anchored proteins in Arabidopsis. A new fellowship in expansion. Plant Physiol. 130, 538–548. 10.1104/pp.007468 12376623 PMC166585

[B70] Schindelman G. Morikami A. Jung J. Baskin T. I. Carpita N. C. Derbyshire P. (2001). COBRA encodes a putative GPI-anchored protein, which is polarly localized and necessary for oriented cell expansion in Arabidopsis. Genes Dev. 15, 1115–1127. 10.1101/gad.879101 11331607 PMC312689

[B71] Sedbrook J. C. Carroll K. L. Hung K. F. Masson P. H. Somerville C. R. (2002). The Arabidopsis SKU5 gene encodes an extracellular glycosyl phosphatidylinositol-anchored glycoprotein involved in directional root growth. Plant Cell 14, 1635–1648. 10.1105/tpc.002360 12119380 PMC150712

[B72] Sedbrook J. C. Kaloriti D. (2008). Microtubules, MAPs and plant directional cell expansion. Trends Plant Sci. 13, 303–310. 10.1016/j.tplants.2008.04.002 18467155

[B73] Seifert G. J. (2021). The FLA4-FEI pathway: A unique and mysterious signaling module related to cell wall structure and stress signaling. Genes (Basel) 12, 145. 10.3390/genes12020145 33499195 PMC7912651

[B74] Seifert G. J. Xue H. Acet T. (2014). The *Arabidopsis thaliana* FASCICLIN LIKE ARABINOGALACTAN PROTEIN 4 gene acts synergistically with abscisic acid signalling to control root growth. Ann. Bot. 114, 1125–1133. 10.1093/aob/mcu010 24603604 PMC4195540

[B75] Shen Q. Bourdais G. Pan H. Robatzek S. Tang D. (2017). Arabidopsis glycosylphosphatidylinositol-anchored protein LLG1 associates with and modulates FLS2 to regulate innate immunity. Proc. Natl. Acad. Sci. U. S. A. 114, 5749–5754. 10.1073/pnas.1614468114 28507137 PMC5465910

[B76] Shi H. Kim Y. Guo Y. Stevenson B. Zhu J. K. (2003). The Arabidopsis SOS5 locus encodes a putative cell surface adhesion protein and is required for normal cell expansion. Plant Cell 15, 19–32. 10.1105/tpc.007872 12509519 PMC143448

[B77] Shpak E. Leykam J. F. Kieliszewski M. J. (1999). Synthetic genes for glycoprotein design and the elucidation of hydroxyproline-O-glycosylation codes. Proc. Natl. Acad. Sci. U. S. A. 96, 14736–14741. 10.1073/pnas.96.26.14736 10611282 PMC24717

[B78] Silva J. Ferraz R. Dupree P. Showalter A. M. Coimbra S. (2020). Three decades of advances in arabinogalactan-protein biosynthesis. Front. Plant Sci. 11, 610377. 10.3389/fpls.2020.610377 33384708 PMC7769824

[B79] Sista Kameshwar A. K. Qin W. (2018). Structural and functional properties of pectin and lignin–carbohydrate complexes de-esterases: A review. Bioresour. Bioprocess. 5, 43. 10.1186/s40643-018-0230-8

[B80] Smyth D. R. (2016). Helical growth in plant organs: Mechanisms and significance. Development 143, 3272–3282. 10.1242/dev.134064 27624832

[B81] Stevens V. L. (1995). Biosynthesis of glycosylphosphatidylinositol membrane anchors. Biochem. J. 310 (Pt 2), 361–370. 10.1042/bj3100361 7654168 PMC1135902

[B82] Takahashi D. Kawamura Y. Uemura M. (2016). Cold acclimation is accompanied by complex responses of glycosylphosphatidylinositol (GPI)-anchored proteins in Arabidopsis. J. Exp. Bot. 67, 5203–5215. 10.1093/jxb/erw279 27471282 PMC5014161

[B83] Tan L. Eberhard S. Pattathil S. Warder C. Glushka J. Yuan C. (2013). An Arabidopsis cell wall proteoglycan consists of pectin and arabinoxylan covalently linked to an arabinogalactan protein. Plant Cell 25, 270–287. 10.1105/tpc.112.107334 23371948 PMC3584541

[B84] Thitamadee S. Tuchihara K. Hashimoto T. (2002). Microtubule basis for left-handed helical growth in Arabidopsis. Nature 417, 193–196. 10.1038/417193a 12000963

[B85] Vaddepalli P. Fulton L. Schneitz K. (2019). Asymmetric redundancy of ZERZAUST and ZERZAUST HOMOLOG in different accessions of *Arabidopsis thaliana* . G3 (Bethesda) 9, 2245–2252. 10.1534/g3.119.400211 31113822 PMC6643898

[B86] Vaddepalli P. Fulton L. Wieland J. Wassmer K. Schaeffer M. Ranf S. (2017). The cell wall-localized atypical beta-1, 3 glucanase ZERZAUST controls tissue morphogenesis in *Arabidopsis thaliana* . Development 144, 2259–2269. 10.1242/dev.152231 28507000

[B87] Verbancic J. Lunn J. E. Stitt M. Persson S. (2018). Carbon supply and the regulation of cell wall synthesis. Mol. Plant 11, 75–94. 10.1016/j.molp.2017.10.004 29054565

[B88] Vogel J. P. Raab T. K. Schiff C. Somerville S. C. (2002). PMR6, a pectate lyase-like gene required for powdery mildew susceptibility in Arabidopsis. Plant Cell 14, 2095–2106. 10.1105/tpc.003509 12215508 PMC150758

[B89] Watanabe Y. Meents M. J. McDonnell L. M. Barkwill S. Sampathkumar A. Cartwright H. N. (2015). Visualization of cellulose synthases in Arabidopsis secondary cell walls. Science 350, 198–203. 10.1126/science.aac7446 26450210

[B90] Wormit A. Usadel B. (2018). The multifaceted role of pectin methylesterase inhibitors (PMEIs). Int. J. Mol. Sci. 19, E2878. 10.3390/ijms19102878 30248977 PMC6213510

[B91] Xiao Y. Stegmann M. Han Z. DeFalco T. A. Parys K. Xu L. (2019). Mechanisms of RALF peptide perception by a heterotypic receptor complex. Nature 572, 270–274. 10.1038/s41586-019-1409-7 31291642

[B92] Xue H. Veit C. Abas L. Tryfona T. Maresch D. Ricardi M. M. (2017). *Arabidopsis thaliana* FLA4 functions as a glycan-stabilized soluble factor via its carboxy-proximal Fasciclin 1 domain. Plant J. 91, 613–630. 10.1111/tpj.13591 28482115 PMC5575511

[B93] Yeats T. H. Bacic A. Johnson K. L. (2018). Plant glycosylphosphatidylinositol anchored proteins at the plasma membrane-cell wall nexus. J. Integr. Plant Biol. 60, 649–669. 10.1111/jipb.12659 29667761

[B94] Yeats T. H. Somerville C. R. (2016). A dual mechanism of cellulose deficiency in shv3svl1. Plant Signal. Behav. 11, e1218108. 10.1080/15592324.2016.1218108 27494413 PMC5155455

[B95] Yeats T. H. Sorek H. Wemmer D. E. Somerville C. R. (2016). Cellulose deficiency is enhanced on hyper accumulation of sucrose by a H+-Coupled sucrose symporter. Plant Physiol. 171, 110–124. 10.1104/pp.16.00302 27013021 PMC4854719

[B96] Zhang Y. Yu J. Wang X. Durachko D. M. Zhang S. Cosgrove D. J. (2021). Molecular insights into the complex mechanics of plant epidermal cell walls. Science 372, 706–711. 10.1126/science.abf2824 33986175

[B97] Zhou K. (2019). Glycosylphosphatidylinositol-anchored proteins in Arabidopsis and one of their common roles in signaling transduction. Front. Plant Sci. 10, 1022. 10.3389/fpls.2019.01022 31555307 PMC6726743

[B98] Zhou K. (2019). GPI-anchored SKS proteins regulate root development through controlling cell polar expansion and cell wall synthesis. Biochem. Biophys. Res. Commun. 509, 119–124. 10.1016/j.bbrc.2018.12.081 30578078

